# Variability analysis of dry spells for improving agribusiness management in Lesotho

**DOI:** 10.4102/jamba.v12i1.814

**Published:** 2020-11-16

**Authors:** Bernard M. Hlalele

**Affiliations:** 1 Department of Business Support Studies, Central University of Technology, Bloemfontein, South Africa

**Keywords:** dry spell, drought, disaster, agribusiness, Lesotho, spectral analysis

## Abstract

In sub-Saharan Africa, rain-fed agriculture remains one of the major sources of food, employment for low-skilled and rural community members and income for both commercial and subsistence farmers. Understanding problems posed by dry spells variability on agribusinesses is one of the critical challenges of our time. This study characterised dry spells in Lesotho for the improvement of agribusinesses using standardised precipitation (SPI) and standardised precipitation evapotranspiration (SPEI) drought indices. This study was found imperative mainly because Basotho’s livelihood is dependent on rain-fed agriculture and this study further aimed to provide an early warning system that could be used for policymaking against adverse effects of drought events in the area. A 30-year-long rainfall and average monthly temperature data were collected from 10 administrative districts of Lesotho and used to compute SPI and SPEI values. Three dry spell parameters – frequency, duration and intensity – were derived from SPI and SPEI time series. The main findings of this study were that all candidate stations experienced similar dry spell conditions in both duration and frequency and all the selected stations throughout the country experienced extreme drought intensity levels from both SPI and SPEI. Two of the 10 districts showed a statistically significant decrease in Mann Kendal’s trend from both SPI and SPEI time series. This implied that farmers must be encouraged to grow drought-resistant cultivars in order to sustain and support agribusiness in Lesotho. Rangeland policies and legislations must be enforced for livestock production, especially in the periods when extreme dry spell events are expected. The government and all other relevant stakeholders are, therefore, encouraged to devise means to support farmers with irrigation systems to maintain agricultural production, revenue and employees’ employment status.

## Introduction

The Economic Research Services of the United States Department of Agriculture (USDA) listed Lesotho amongst the countries where approximately 100% of the population is projected to remain food insecure until 2022 (United States Department of Agriculture 2012). This food insecurity phenomenon has resulted in 80% of the population of this country being hungry because their livelihood means depend on rain-fed agriculture. Given this kind of livelihood dependence, the key threats to development in this country are chronic poverty and a high unemployment rate, to mention but a few, exacerbated by a decrease in remittances from migrant labourers in South Africa because of the closure of mines and climate-related shocks such as frequent droughts (United Nations World Food Programme (WFP) 2015). However, agriculture, which is the main source of livelihood, is hampered by the following: lack of arable land because of increased urbanisation, soil erosion and environmental degradation, an unfavourable climate that exacerbates and increases drought frequency and over-reliance on rain-fed subsistence agriculture (Assessment Capacities Project (ACAP) 2013).

Lesotho is known for its unique and rich cultural practices, such as the initiation of schooling [*Lebollo*] for both boys and girls. This practice, which is intended to initiate boys and girls into manhood and womanhood, respectively, lasts approximately for 6 months where the initiates are constantly engaged in collecting large amount of firewood (Matobo, Makatsa & Obioha 2009). In addition to this practice, the bulk of domestic energy consumption in Lesotho is based on using wood as fuel. Although there is very limited tree cover in Lesotho, the majority of people rely on wood as a source of energy (United Nations Environment Programme (UNEP) 2013). Limited tree cover and cutting of trees by households for fuel purpose expose the land to an increasing number of drought events. Land-use management is the direct responsibility of local chiefs at village level, some of whom are illiterate. There are also poor land policies and enforcement of management measures, which lead to land degradation, hence increased vulnerability to drought impacts (Maro 2011).

Given that 80% of population’s livelihood depends on rain-fed agriculture, Lesotho is currently facing the following problems: (1) high unemployment and chronic poverty levels (WFP 2015); (2) lack of arable land because of increased urbanisation, soil erosion and environmental degradation (ACAP 2013); (3) a high deforestation rate as a result of cultural practices [*Lebollo*] (Matobo et al. 2009); (4) wood as the main source of fuel (UNEP 2013); (5) illiterate local chiefs charged with direct responsibility of land-use management (Maro 2011); (6) poor policies and management measures against droughts (Maro 2011); and (7) no drought risk reduction-specific framework to manage drought disaster risks given that 80% of the population depends on rain-fed agriculture. Drought disaster issues are, therefore, addressed haphazardly, with no guiding principles, leading to poor planning and hence unpreparedness that leads to a reactive disaster management approach. This situation adversely does not only affect peoples’ livelihood but also the agribusinesses.

## Socio-economic impacts of drought

Drought is the most complex of all natural disasters with damaging and severe impact on agricultural production, ecosystems, water resources and society. This complexity makes the detection of its start and end periods difficult (Tan, Yang & Li 2015). This is directly linked with precipitation, the lack of which affects various economic sectors, leading to substantial costs for communities (Haensel, Matschullat & Schucknecht 2015). However, the term ‘drought’ has a larger number of definitions drawn from different perspectives and perceptions with regard to the purpose that is defined (Gregor 2013). This natural phenomenon has various impacts, ranging from direct to indirect, and from different dimensions, as shown in Table 1. Historically, drought has globally caused both direct and indirect economic, social and environmental problems, some of which are inevitable even with early preparations (UCAR 2015). Similarly, Anon (2015) asserts that this natural hazard produces a complex web of impacts that span over several sectors of the economy. This complexity is because of the lack of water, which is integral to communities in producing their own food and services. Despite the social, economic and environmental adverse impacts, droughts pose difficulty in decision-making with regard to water allocation and result in stringent water-use limitations (National Drought Policy Commission 2015). Hazards related to climate and weather, such as drought, affect more people and have larger economic damage worldwide than any other type of hazards; these hazards have killed or affected 70 times as many people and caused twice as much damage worldwide as did any other hazard types (Arnold & Kreimer 2000).

**TABLE 1:** Direct and indirect impacts of drought.

Aspect/dimension	Direct impacts	Indirect impacts
Environmental	Soil moisture	Water quality
	Groundwater level	Biomass development
	Runoff	Biodiversity
	Springs’ yields	Dust storms
	Surface runoff	Desertification
	Water level in lakes	Forest fires
	Available (exploitable) amounts of drinking water	
Economic	Exploitation of surface water	Irrigation water
	Exploitation of groundwater	Water for farming
	Diminishing of drinking water sources	Failure of irrigation
		Loss of animals on farms
		Reduction of navigable rivers
		Reduce of hydroelectric power
		production
		Food prices increasing
		Reduction of economic growth
Social	Drinking water	Conflicts and conflicts of interest

*Source*: Gregor, M., 2013, ‘Principles of drought analysis and assessment’, *Water International* 4(3), 1–53

One of the biggest weather hazards, which many people hardly realise, is heat that kills many people silently (NOAA 2015). Globally, the number of weather-related natural disasters has more than tripled since the 1960s. These disaster events claim over 60 000 lives annually in developing countries (WHO 2015). Extreme high air temperatures directly cause cardiovascular and respiratory diseases, especially amongst the elderly and the young: groups of over 65 and younger than 5 years, respectively. A number of people feel comfortable with temperature in the ranges of 20 °C – 27 °C, and relative humidity of 34% – 60%; however, as these temperatures go higher, the body’s coping mechanism becomes overwhelmed, leading to various and possibly fatal conditions (Canadian Center for Occupational Health and Safety 2015). Some of the disorders caused by heat are sunburn, heat cramps, heat exhaustion and heat stroke (NOAA 2015). Drought ranks highest amongst all natural disasters globally in terms of economic impacts and causes 78% of other natural disasters (DIMTEC 2015; Geerts & Linacre 2015).

Finally, drought and its impacts are really two sides of the same coin. We cannot fully understand drought without understanding its impacts, which can affect all parts of our environment and our communities. Understanding drought conditions, societal vulnerability and their related effects on one another provides us with historical lessons that can aid in dealing with future drought conditions (NOAA 2015).

The following countries shown in Table 2 were identified by the World Bank as the most at risk of drought in 2009. Therefore, this study reviewed the impacts from economic, environmental and social spheres.

**TABLE 2:** Impacts of drought in Asian countries.

Country	Drought impacts		
	Economic	Environmental	Social
India	$95.4 million incurred on water supply tankers and repairs of existing water systems in March 2013, 21%, 5% and 18% reduction in cereals, pulses and total food grains production, respectively, for the year 2012–2013 as compared to the previous year, 33% and 29% reduction in sugarcane and citrus fruit production, respectively, 11% decrease in vegetables production in 2012 compared to 2013, $84.5 million of drought mitigation strategies in implementing 441 cattle camps, farmers forced to borrow money from money lenders and banks with high interest rates	Water scarcity in the state of Maharashtra, 1 m decline in groundwater level.	The social life and mental health of farmers and others in the drought affected rural communities, hopelessness and mental depression because of the adverse impacts of drought. There is an abnormally high rate of farmer suicide in the state, and in India as a whole, because of lack of social and community support in the existing drought relief packages.
Iran	Increase in costs of labour and weed removal, increase in costs for water supply, decrease in purchasing power, decrease in savings, non-payment of bank loans and obligations, increase in the false financial relationship, decrease in price of crops because of reduction in quality, decrease in income because of reduction of cultivation, decrease in land price, decrease in income from side jobs.	Decrease in river flow and groundwater levels, decrease in surface water reservoirs and ponds, increase in weeds growing in fields, increase in mortality of fish and other aquatics in ponds, decrease in water quality, increase in pest attacks, increase in plant diseases, increase in soil erosion, increase in amount and intensity of fires, decrease in diversity of plant species.	Increase in frustration, anxiety and emotional problems, feelings of poverty and decrease in life level, decrease in recreational activities, increase in local divisions to supply water, weakened position of institutions and cooperative unions, weakened traditions of cooperation, increase in tendency to migrate, decrease in social ceremonies, decrease in the level of education of children and juveniles, disintegration of consistency and continuity in family systems.

*Source*: Golmohammadi, F., Arazmjoo, M. & Razavi, S.H., 2012, ‘Investigating importance and effects of climate changes in agriculture in South Khorasan Province and recognizing appropriate extension education activities in confronting them’, *International Conference on Applied Life Sciences*, pp. 381–386

Disasters reverse development progress by years, sometimes even by decades, and leave the affected countries with monumental debts for projects that were destroyed. Therefore, development organisations responsible for recovery projects must consider an effort to increase local resilience (Coppola 2011). Table 3 presents the impacts of drought in the greater Horn of African countries that are at risk of drought hazard.

**TABLE 3:** Drought hazard occurrence and impact of damage.

Country	Frequency of occurrence	Drought impacts
Djibouti	Several droughts over the years (1980, 1996, 2001, 2005, 2008)	Since 2007, agriculture and rural livelihoods of nearly 50% of the rural population (120 000 people), approximately 15% of the total population, have been affected.
Ethiopia	At least five major national droughts since 1980	About 11% of the total population exposed to droughts, mainly pastoral areas.
Kenya	Major droughts every 10 years and minor ones almost every 3–4 years.	Between 1983 and 1993, droughts in the ASALs have become longer and more frequent, resulting in significant loss of agricultural production.
Somalia	Devastating droughts happened during 1963–1964, 1974–1975 and recently in 2011.	Between 2010 and 2012, more than 258 000 people died – half of the victims were children younger than 5 years.
South Sudan	The worst drought hit during 1980–1984 and 2011.	Widespread displacement and localised famine in some parts of the country.
Sudan	Most serious drought incidents were in 1970, 1983–1985, 1991–1992 and 2010–2011.	The 1983–1985 and the 2010–2011 droughts resulted in mass deaths of human and livestock.
Uganda	There were seven droughts between 1991 and 2000 with increased frequency. There were recent droughts in 2008 and 2013.	Karamoja region in 1991–2007 had severe droughts, leading to depletion of pasture and severe lack of water for livestock, intensifying conflicts.

*Source*: Global Water Partnership Eastern Africa (GWPEA), 2015, *Assessment of drought resilience frameworks in the Horn of Africa*, Integrated Drought Management Program in the Horn of Africa (IDMP HOA), Entebbe

Once the impacts are known, it is the responsibility of every country to implement preventative measures. Lessons learnt from those who have experienced such disasters are needed to guide measures for implementation.

## Methods and materials

This study followed a quantitative method research design where a researcher relies on numeric data in testing relationships between variables (Creswell et al. 2007). They assert that the researcher also relates the variables to determine the magnitude and frequency of relationships. Moreover, the ultimate goal of quantitative research is to describe the trends, if any exist, or explain relationships between or amongst variables. This study used the positivist research paradigm.

## Data collection

**FIGURE 1:**
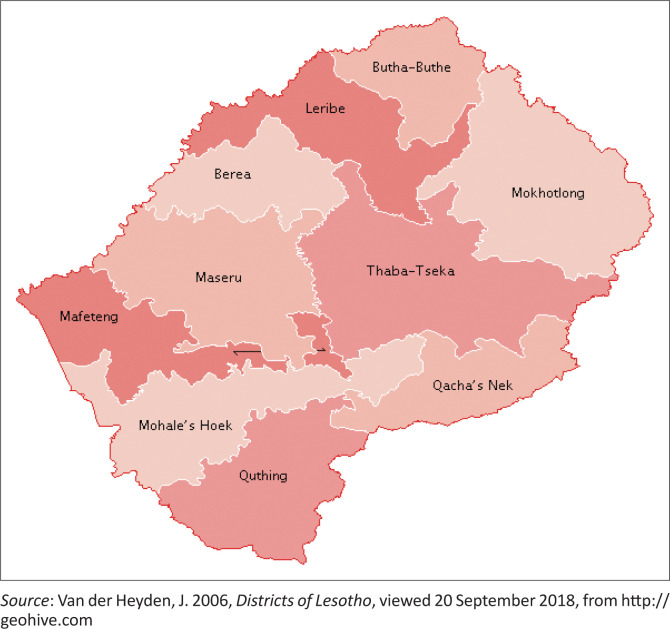
Administrative divisions map of Lesotho, showing 10 districts.

Data for this study were collected from at least one weather station from each of the 10 administrative districts of Lesotho or a nearby station. Data collection was mainly focused on monthly precipitation and temperature data from the past 30 years (1985–2015) over all the identified weather stations, with complete available data in Lesotho. The Lesotho Meteorological Services and Water Affairs are the responsible government departments for keeping the meteorological and stream flow data in Lesotho, respectively. Therefore, the two sets of data (precipitation and temperature) were collected from the Lesotho Meteorological Services and Water Affairs. Figure 1 shows the map of Lesotho exhibiting the 10 districts that were used as selected stations from which data were collected for this study.

All stations from which data were completely missing were exempted from participation in the study. However, in the stations where at least 10% of data were missing, gaps were filled by the methods discussed below. In literature, there exist several techniques for estimating missing data. Two of these techniques are discussed and the discussion ends with the one used in the study.

Expectation Maximum (EM) is defined as a statistical algorithm suitable when there are missing or hidden values in the data sets (Hauskrecht 2017). Borman (2006) adds that EM is a popular tool used in statistical estimation problems that involve incomplete data. Similarly, Chuong and Serafim (2008) refer to EM as an algorithm that enables parameter estimation in probabilistic models with incomplete data. Prior to missing values estimation, data collected from the Lesotho Meteorological Services and Water Affairs were compared for quality, reliability and validity. The EM was, therefore, used in the study. Both monthly values of temperature and precipitation were entered into an IBM Statistical Package for the Social Sciences (SPSS) v. 24 where the EM algorithm was applied to estimate all missing values in the data sets. The three complete data sets were then subjected to outliers detection aided by the IBM SPSS v24 program. Prior to any climatological data analysis, data sets must be tested for homogeneity.

The selected indices, SPI and SPEI, were calculated from DrinC computer software. The SPEI that uses precipitation and potential evapotranspiration, was computed from the drought calculator (DrinC) using Hargreaves methods, which use both minimum and maximum temperature values. DrinC is an open access software developed for calculating drought indices such as SPI. Two indices, SPI and SPEI, were then computed to detect drought conditions from only a 3-month time scale. Standardised precipitation-3 was used because it measures agricultural drought on seasonal basis. The dry spell parameters drawn from duration, intensity and frequency were calculated from a customised computer program. These parameters included the number of dry spell events (*N*) and average dry spell duration, average dry spell intensity (ADSI) and the ratio of number of dry spell events (%) during the 30-year study period (*N*/30) for frequency. The four dry spell parameters drawn from duration, intensity, frequency and station elevation were in the *k*-means clustering of stations on temporal scales. This resulted in regions ranked for easy management, planning and prioritisation. Standardised precipitation intensity maps were then generated and displayed through a StatPlanet computer program.

Prior to the above analysis, homogenisation was employed. Homogenisation has to be undertaken prior to any data analysis as part of data quality control measures in order to eliminate any erroneous and non-climatic biases in the time series (Štěpánek et al. 2013). Most climatological time series suffer from in-homogeneities because of (1) changes in instrument settings, (2) changes in observers, (3) changes in formula calculations, (4) changes in observer practices and (5) station relocations. Homogeneity is an important issue in climate change data analysis to detect variability in the data series. This generally means that when data are homogeneous, they were taken at the same time with the same instruments and in the same environments. In this way, homogenisation of time series data ensures reliability of the results (Franz, Steffan-Dewenter & Menzel 2009). Before any analysis begins, exploratory data analysis must be conducted. Its advantage is to provide a preliminary indication of trends present in the data that enable further analysis (Meals et al. 2011). The authors further emphasise that this technique requires fairly long series of data, collected by consistent methods with few gaps. Most trends and analysis procedures require certain assumptions, such as the data must follow a certain probability distribution (Xie et al. 2016). A non-parametric homogeneity test was, therefore, used in monthly precipitation and minimum and maximum temperature.

### Ethical consideration

This article followed all ethical standards for a research without direct contact with human or animal subjects.

## Results and discussions

### Homogeneity test results

In statistics, homogeneity tests are conducted to examine statistical properties of a particular data set. It actually examines the location stability and local fluctuations in the time series over time (Spider Financial Corp 2012). The author asserts that this phenomenon is equivalent to testing statistical distribution, hence detecting if any changes in the distribution exist. The test is conducted to avoid spurious results from the data sets. Table 4 presents a homogeneity test over all 11 selected stations in minimum and maximum temperature and precipitation. A non-parametric Pettitt’s test was used. In all stations, a two-tailed hypothesis was used in all the three input parameters, where all the data sets are homogeneous with *p*-values all greater than a specified significant level of 0.05. This takes the study to the next level of exploration and further analysis. This implication for homogeneity test results in Table 4 is that all subsequent analyses will be free from errors, thereby reflecting the true characteristics of the station and its surroundings.

**TABLE 4:** Non-parametric homogeneity test (Pettitt’s test).

Station	Elevation (m)	Pettitt’s test at 5% significant level								
		*T* min (°C)			*T* max (°C)			Preci (mm)		
		*k*	*t*	*p* (two-tailed)	*k*	*t*	*p* (two-tailed)	*k*	*t*	*p* (two-tailed)
Butha Buthe	1770	2 598 000	2003	0.543	1 929 000	2003	0.093	2 661 000	2003	0.582
Leribe	1740	1 204 000	1993	0.061	2 902 000	1988	0.798	3 932 000	2001	0.469
Mafeteng	1610	2 937 000	1996	0.828	2 830 000	2006	0.750	3 313,000	2009	0.865
Mejametalana (Maseru)	1530	2 597 000	2003	0.544	2 613 000	1990	0.545	4 325 000	1995	0.317
Mohale’s Hoek	1620	19 538 000	1985	0.080	20 729 000	2014	0.056	11 985 000	1987	0.356
Mokhotlong	2230	2 730 000	1996	0.652	3 650 000	2002	0.643	3 362 000	1995	0.822
Oxbow	2600	4 264 000	1999	0.335	4 265 000	1997	0.334	2 509 000	2000	0.462
Qacha’s Nek	1970	1 762 000	2002	0.055	4 619 000	2001	0.216	5 322 000	1995	0.087
Quthing	1740	3 205 000	1987	0.957	1 897 000	1998	0.082	4 513 000	1995	0.255
Semonkong (Maseru)	2458	2 439 000	1996	0.410	2 983 000	2008	0.875	7 930 000	2000	0.063
Thaba Tseka	2160	3 005 000	1997	0.893	2 630 000	1990	0.560	2 448 000	1995	0.410

### Mann-Kendall’s trend analysis of standardised precipitation evapotranspiration/standardised precipitation

**FIGURE 2:**
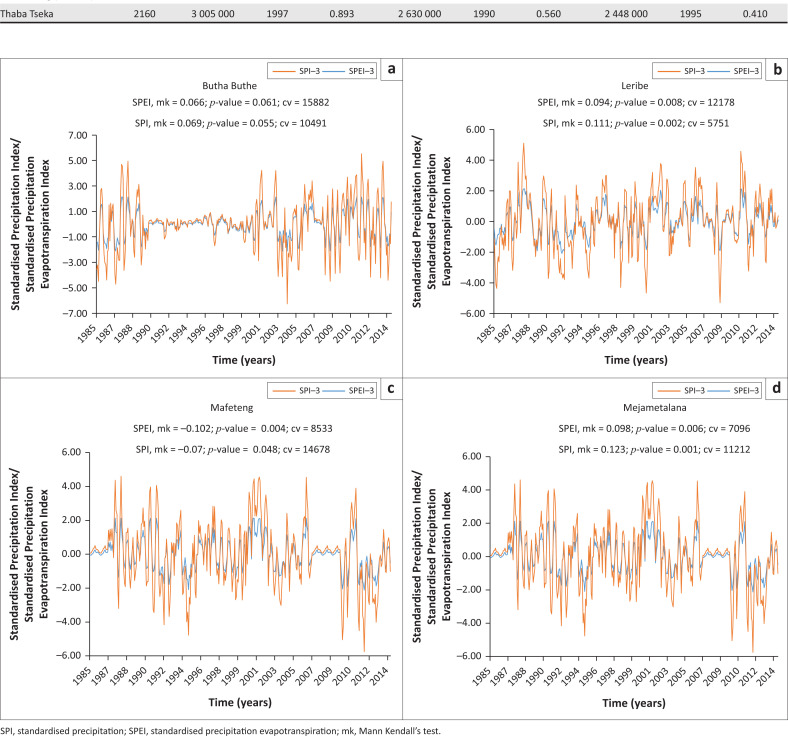

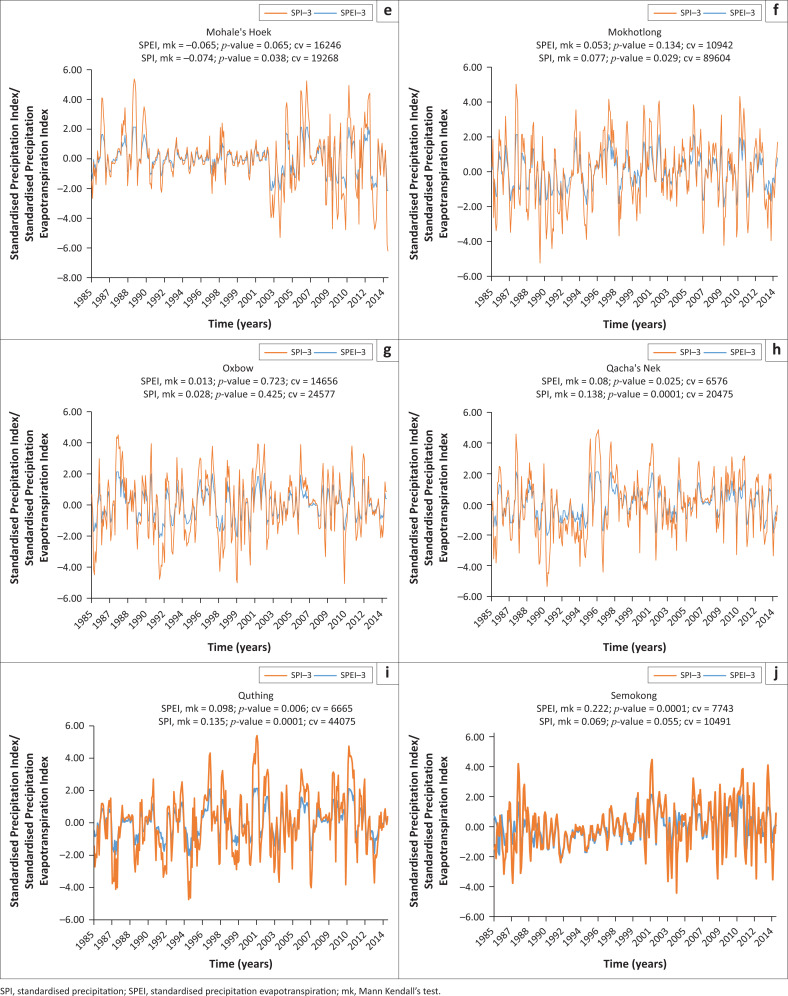

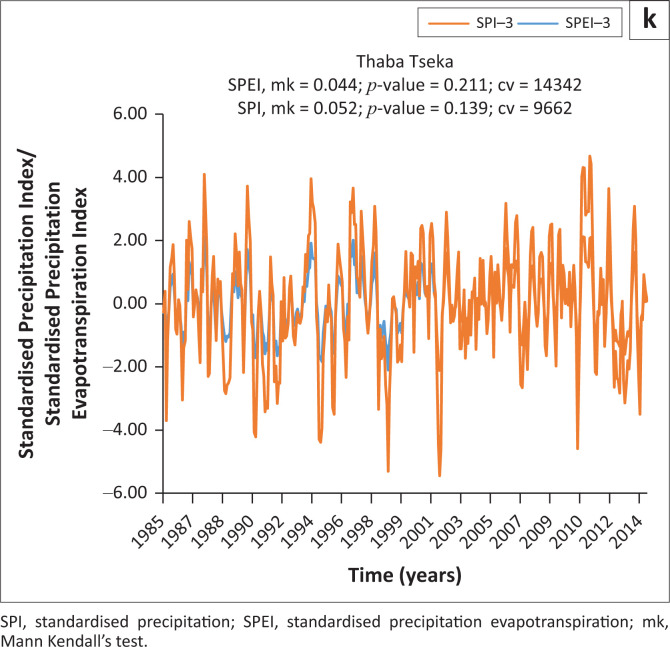
Standardised precipitation/standardised precipitation evapotranspiration-3 plot. FIGURE 2 continues on the next page →

Figure 2a–k shows the plots of the SPEI and SPI on a 3-month time scale. These two indices show almost the same behaviour in terms of trends across the 11 stations. Both indices showed significant decreasing patterns at the Mafeteng station. However, the SPI-3 again indicates Mohale’s Hoek as having a decreasing pattern. The SPI-3 therefore picks more significant trends than the SPEI-3. Moreover, the coefficient of variation in the SPI is twice as much as that of the SPEI. Other stations showed neither decreasing nor increasing trends in the 3-month time scale significant at < 0.05. An increase in dry spells implies better drought conditions as values move from negative to positive. The SPI showed all stations with significant trends compared with the SPEI, and with a greater coefficient of variation (CV). This brings the SPI to pick dry and wet spell better and way before the SPEI across the selected time scales. At this point the SPI seems to outsmart the SPEI; however, the two indices were only compared regarding dry spell parameters in duration, intensity and frequency.

**FIGURE 3:**
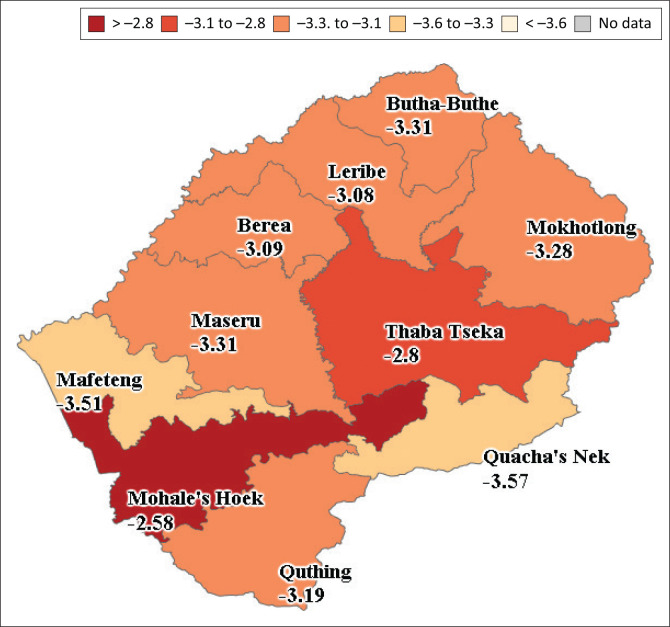
Dry spell spatiotemporal standardised precipitation-3 intensity maps (1985–2014).

Table 5 shows SPEI-3 and SPI-3 duration, intensity and frequency parameters over all the selected stations in Lesotho. It can be seen that these two drought indicators performed almost the same way across the stations. This implies that SPI, which is a single input drought indicator, is sufficient in quantifying droughts. Figure 3 depicts the spatial extent of agricultural drought in the study area, where all regions were found to be in extreme drought category.

**TABLE 5:** Standardised precipitation evapotranspiration and standardised precipitation drought parameters.

Station	SPEI-3				SPI-3			
	Duration		Intensity ADSI	Frequency (*N* = 30)	Duration		Intensity ADSI	Frequency (*N* = 30)
	*N*	ADSD			*N*	ADSD		
Butha Bothe	53	3.11	−2.42	177	37	3.43	−3.31	123
Leribe	48	3.83	−2.66	160	43	3.70	−3.08	143
Mafeteng	41	4.15	−3.05	137	38	3.95	−3.51	127
Mejametalana	48	3.58	−2.63	160	45	3.36	−3.09	150
Mohale’s Hoek	50	3.84	−2.50	167	45	3.76	−2.58	150
Mokhotlong	44	4.02	−2.87	147	43	3.81	−3.28	143
Oxbow	45	3.93	−2.82	150	42	3.62	−3.34	140
Qacha’s Nek	42	4.17	−2.98	140	39	4.26	−3.57	130
Quthing	42	4.00	−3.00	140	43	3.88	−3.19	143
Semonkong	36	4.89	−3.49	120	37	3.43	−3.31	123
Thaba Tseka	48	3.71	−2.66	160	48	3.35	−2.80	160

ADSD, Average Dry Spell Duration; ADSI, average dry spell intensity; SPI, standardised precipitation; SPEI, standardised precipitation evapotranspiration.

## Conclusion and recommendations

Rainfall is the most critical and key variable for both hydrological and atmospheric circles, the lack of which leads to extremes such as droughts. The awareness of the characteristics of dry spells over an area, such as source, intensity, duration, variability, distribution and frequency, is essential for proper and efficient control and management of water resources (Takele & Gebretsidik 2015). On SPI-3, Mohale’s Hoek was the only station that showed a statistically significant decreasing trend. Standardised precipitation detected dry spells much earlier than SPEI over all stations, showing a higher sensitivity than SPEI. This situation leads to SPI being the most suitable index for dry spells analysis in the study area. All analyses that followed used SPI only because of its sensitivity to pick up dry spells earlier than a water balance index (SPEI). All stations showed a high and long frequency and duration, respectively. Moreover, the entire study area was in extreme drought during the study period. All stations were under extreme drought, which indicates that the situation of drought condition is yet to stay constant over years to come. This implies that farmers must be encouraged to grow drought-resistant cultivars to keep agribusiness in the market. Rangeland policies and legislation must be enforced for livestock production, especially in the periods when drought events are expected. It is, therefore, recommended that the agricultural sector should remain vigilant at all times as drought episodes may strike at any given period.
